# The Value of Buttermilk in Disease

**Published:** 1908-08-15

**Authors:** 


### Diseases of Children.

### THE VALUE OF BUTTERMILK IN DISEASE.

Considerable attention has been paid to the bacteriological flora of the intestinal tract in health and disease. It has been found that the lactic acid bacillus is almost always present, often in huge numbers. Apparently this organism grows with the utmost freedom, and either crowds out other bacteria or is inimical to their growth by virtue of the lactic acid which is produced. On these grounds buttermilk or tablets composed of lactic acid bacilli have been advocated for the treatment of gastric and intestinal affections, which depend on putrefactive changes set up by proteolytic organisms.

Buttermilk can be made from sour cream or from milk which has been previously skimmed. For intestinal affections it is best made from sour skimmed milk, by churning one pint at a temperature of 60° F. to 70° F. for fifteen minutes, in a churn of two pints capacity. The milk should be as fresh as possible, not more than 24 hours old in hot weather. On the whole it is even better to obtain fresh centrifugalised, or whole milk, and heat it to a temperature of 170° F., in order to destroy any deleterious organisms which may be present, to add lactic acid bacilli, keeping the mixture at the required temperature until sour, and then to churn it. When it has been made it should be stood on ice or in a refrigerator, and used during the next 24 hours. In the process of preparation the milk-casein is finely divided by the churning, and separated from its calcium base. The calcium is transformed into soluble salts, chiefly the lactate, which amounts to about 1.85 grammes in the litre.

Salge states that when made from sour cream it contains the following percentages of the food constituents:—Proteid, 2.5 to 2.7; fat, 0.5 to 1.0; sugar, 3.0 to 3.5. The decrease in the percentage of sugar from that normally present in milk is due to lactic acid fermentation. The acidity is about 0.5 per cent., and the calorie value 300 to 450 per litre. The food can be fairly well taken raw, but it is more usual to sweeten and sterilise it. Sometimes it is mixed with 10 to 25 grammes of flour, and 35 to 90 grammes of sugar per litre, and brought to a boil three times. Or a teaspoonful of wheaten flour and four of granulated sugar are stirred into a quart of the buttermilk, and the mixture is brought to a boil in a double saucepan, with frequent stirring. Care must be taken not to curdle it by too much heat, or the divided casein will be found to clot into large masses like ordinary sour milk. This can generally be prevented by constant stirring, or by the addition of carbonate of soda up to slight alkalinity.

A ready means of curdling the milk is by the addition of the "Sauerin" tablets of lactic acid bacilli, supplied by Allen and Hanburys, or "Lactobacilline" powders and tablets prepared in Paris according to the directions of Metchnikoff, and sold in this country by Wilcox, Jozeau and Co. The latter firm also supply a lactobacilline broth made from malt, suitable for cases in which it is inadvisable to give milk in any form.

Another preparation deserving of mention is a condensed variety sold under the name of Condensed Buttermilk "Lactitia." Its composition is given by the makers as: Albumen, 3.02; fat, 0.89; milk sugar, 2.71; invert and cane sugar, 4.99; meal, about 1.00; with a calorie value of 590 per litre. The child can be fed entirely on this food, and it has proved very useful in some cases of malnutrition, marasmus, enteric catarrh and ileo-colitis. The stools assume a yellowish tinge and are not unduly frequent. It is not always successful, especially under six months of age, and it may cause vomiting.

It is obvious that in the above preparations we are dealing with very different substances, according to the way in which they are made. In some there is a liberal supply of lactic acid, and in others there is in addition a huge number of active bacilli. Lactic acid has been used for very many years in the treatment of infantile diarrhœa, and the writer remembers advocating its use in this complaint quite 10 to 15 years ago. It is undoubtedly of value in certain mild cases. But it is to the free administration of active bacilli we should direct our thoughts. There is evidence in favour of the view that these organisms can produce a profound alteration in the intestinal contents in the course of a few days to a week. The reaction becomes neutral or slightly acid, and consequently much less suitable for the growth of putrefactive organisms. By this means intestinal antisepsis is brought about in a manner not deleterious to the system, and free from the risks of most antiseptic drugs, which are rarely of any value unless given in dangerous doses, with the exception of insoluble ones such as $\beta$-naphthol. In addition, we must bear in mind that another factor comes into play, namely, the withdrawal of fat from the food. Atrophic infants digest the fat of cows' milk badly, and it is not well borne in cases of intestinal disorders in infancy. Possibly, therefore, some of the advantages derived from buttermilk feeding are due to the deficiency of fat in the food. It is an important point to bear in mind, for it indicates that the food must not be continued for long at a time, seeing that it is so deficient in a most necessary article of the infantile dietary. For older children and adults there is yet another way in which we can make use of lactic acid bacilli. They can be given in the form of tablets or powders. When so used, it is advisable to give a purely milk diet, skimmed if necessary, and to give one of the tablets after each dose of milk. Recently, in a case of chronic ulcerative colitis thus treated, recovery was most rapid. On the other hand, in a milder case in which there was evidence of only a limited patch of ulcerative colitis in a child treatment was apparently valueless.

At present the treatment must be regarded as on its trial, though it may justly be stated that in the experience of the writer it has sometimes proved of undoubted benefit. It must be used with judgment and discretion, not persisted in for long unless distinctly beneficial, and not regarded as a cure for every alimentary disorder. Kephir and koumiss are closely analogous preparations.

